# Low-Level Lead Exposure and Elevations in Blood Pressure during Pregnancy

**DOI:** 10.1289/ehp.1002666

**Published:** 2011-02-03

**Authors:** Ellen M. Wells, Ana Navas-Acien, Julie B. Herbstman, Benjamin J. Apelberg, Ellen K. Silbergeld, Kathleen L. Caldwell, Robert L. Jones, Rolf U. Halden, Frank R. Witter, Lynn R. Goldman

**Affiliations:** 1 Department of Environmental Health Sciences, Case Western Reserve University School of Medicine, Cleveland, Ohio, USA; 2 Department of Environmental Health Sciences, Johns Hopkins Bloomberg School of Public Health, Baltimore, Maryland, USA; 3 Department of Epidemiology, Johns Hopkins Bloomberg School of Public Health, Baltimore, Maryland, USA; 4 Columbia Center for Children’s Environmental Health, Columbia University Mailman School of Public Health, New York, New York, USA; 5 Division of Laboratory Sciences, National Center for Environmental Health, Centers for Disease Control and Prevention, Atlanta, Georgia, USA; 6 Center for Environmental Biotechnology, Biodesign Institute, Arizona State University, Tempe, Arizona, USA; 7 Department of Gynecology and Obstetrics, Johns Hopkins University School of Medicine, Baltimore, Maryland, USA; 8 George Washington University School of Public Health and Health Services, Washington, DC, USA

**Keywords:** benchmark dose, blood pressure, hypertension, lead, pregnancy, risk assessment, umbilical cord

## Abstract

**Background:**

Lead exposure is associated with elevated blood pressure during pregnancy; however, the magnitude of this relationship at low exposure levels is unclear.

**Objectives:**

Our goal was to determine the association between low-level lead exposure and blood pressure during late pregnancy.

**Methods:**

We collected admission and maximum (based on systolic) blood pressures during labor and delivery among 285 women in Baltimore, Maryland. We measured umbilical cord blood lead using inductively coupled plasma mass spectrometry. Multivariable models were adjusted for age, race, median household income, parity, smoking during pregnancy, prepregnancy body mass index, and anemia. These models were used to calculate benchmark dose values.

**Results:**

Geometric mean cord blood lead was 0.66 μg/dL (95% confidence interval, 0.61–0.70). Comparing blood pressure measurements between those in the highest and those in the lowest quartile of lead exposure, we observed a 6.87-mmHg (1.51–12.21 mmHg) increase in admission systolic blood pressure and a 4.40-mmHg (0.21–8.59 mmHg) increase in admission diastolic blood pressure after adjustment for confounders. Corresponding values for maximum blood pressure increase were 7.72 (1.83–13.60) and 8.33 (1.14–15.53) mmHg. Benchmark dose lower limit values for a 1-SD increase in blood pressure were < 2 μg/dL blood lead for all blood pressure end points.

**Conclusions:**

A significant association between low-level lead exposures and elevations in maternal blood pressure during labor and delivery can be observed at umbilical blood lead levels < 2 μg/dL.

Hypertension is a major risk factor for future cardiovascular disease (Chobanian et al. 2003; Greenlund et al. 2004). It has also been demonstrated that elevated blood pressures (between normal and clinically hypertensive) are indicative of increased cardiovascular risk (Kshirsagar et al. 2006). Pregnancy poses additional risks from high blood pressure. Hypertension occurring during pregnancy may lead to preeclampsia, a serious condition associated with substantial maternal and fetal morbidity and mortality (Solomon and Seely 2006). Hypertension during pregnancy may also predispose women to development or increased severity of future cardiovascular disease (Garovic and Hayman 2007; Moreira et al. 2009).

Well-established risk factors for hypertension include age, race, obesity, diet, and family history (Whelton et al. 2002). Primiparity and multiple gestations are additional risk factors for gestational hypertension (GH) and preeclampsia (Solomon and Seely 2006). In addition, there is evidence that lead exposure in adults is a risk factor for elevated blood pressure (Navas-Acien et al. 2007; Nawrot et al. 2002). Lead has also been associated with GH (Chen et al. 2006; Magri et al. 2003; Rabinowitz et al. 1987; Rothenberg et al. 2002; Vigeh et al. 2004; Yazbeck et al. 2009) and/or preeclampsia (Dawson et al. 2000; Sowers et al. 2002; Vigeh et al. 2006), as well as elevated blood pressure (Magri et al. 2003; Rabinowitz et al. 1987; Rothenberg et al. 1999, 2002).

However, most of these studies were conducted in populations with lead levels substantially higher than current levels in the United States and other developed countries, where blood lead levels in all age groups have markedly declined primarily due to the banning of lead in gasoline and other regulatory actions initiated to limit lead exposure [Centers for Disease Control and Prevention (CDC) 2009; Meyer et al. 2003]. Recent evidence demonstrates that despite this general decline in population lead exposure, higher lead blood levels remain positively associated with hypertension, particularly among adult African Americans and Mexican Americans (Muntner et al. 2005). Yazbeck et al. (2009) have shown that low blood lead concentrations are related to GH. The goal of the present study was to evaluate the relationship of lead as measured in umbilical cord blood with maternal blood pressure during pregnancy, within the Baltimore Tracking Health Related to Environmental Exposures (THREE) Study.

## Methods

### Study population

We performed a cross-sectional study of 285 births from the Baltimore THREE Study. Details about this study have been published previously (Apelberg et al. 2007). This study had the approval of the Maternal and Fetal Research Committee, Department of Gynecology and Obstetrics, and the Johns Hopkins School of Medicine Institutional Review Board (IRB). We obtained a Health Insurance Portability and Accountability Act waiver. The IRB waived the requirement for informed consent because the collected biological samples would otherwise have been discarded and the sample collection constituted no more than minimal risk to the subjects. Strict procedures protected subject confidentiality, and all data and specimens were anonymized.

There were 615 births (603 deliveries) between November 2004 and March 2005. Of these, we excluded multiple births (*n* = 24 infants) and births where cord blood was unavailable or of insufficient quantity (*n* = 291), leaving a total of 300 births eligible for the study. These are representative of all births at the hospital, except that there were fewer low-birth-weight infants because smaller babies tended to have less cord blood. Fifteen infants had missing data for key variables (*n* = 13) or for having outlying data (*n* = 2), and we excluded them from analyses. The two outliers included one subject with an admission systolic blood pressure (SBP) < 20 mmHg (likely due to measurement or recording error) and one with an umbilical cord blood lead value of 15.5 μg/dL (more than two times higher than the second largest value). Sensitivity analyses confirmed that the exclusion of the lead outlier did not substantially alter results.

### Data collection

Two study personnel collected data from maternal medical records. Study clinicians reviewed a 10% random sample for accuracy.

### Blood pressure end points

From the medical record we collected diagnoses of GH, preeclampsia, chronic hypertension, and use of antihypertensive medications. We created two variables to summarize these: *a*) those with GH or preeclampsia versus not, and *b*) those with GH, preeclampsia, or chronic hypertension or used hypertensive medications (referred to as “any hypertension”) versus not. Hospital personnel measured maternal blood pressure at admission for labor and delivery and continuously during hospitalization using a General Electric Corometrics model 120 series fetal monitor (GE Healthcare, Little Chalfont, UK), which uses a noninvasive blood pressure measurement based on the DINAMAP algorithm. The monitor measured blood pressure at programmed intervals ranging from once per minute to once per hour, depending on clinical needs. The varying time intervals, combined with the fact that women were in the hospital for varying amounts of time, resulted in large variability in the quantity and frequency of available measurements. We recorded three pairs of blood pressure measurements from each mother: SBP and diastolic blood pressure (DBP) at admission, the maximum SBP and corresponding DBP, and the minimum SBP and corresponding DBP. We compared these measurements with relevant characteristics from the medical record to validate their utility. Blood pressure measurements were not related to the time of day of hospital admission. Those with a hypertension diagnosis or using antihypertensive medications had higher admission and maximum blood pressure measurements than did mothers without any hypertension (Student’s *t*-test, *p* < 0.05). Minimum blood pressure was only weakly associated with measures of hypertension and was also the only blood pressure measurement significantly related to length of labor and type of delivery [Spearman correlation, *p* < 0.05; one-way analysis of variance (ANOVA), *p* < 0.05, respectively]. Moreover, our observations suggest that minimum blood pressure measurements may be influenced by events during labor and delivery such as a blood pressure cuff being too loose for an accurate measurement or use of pain reduction medication and therefore were not used in analysis.

### Umbilical cord blood collection and lead measurements

Trained clinical staff used a well-defined method to collect umbilical cord blood immediately after the cord was cut (Witter et al. 2001). After temporary storage (< 3 hr) at 4°C, we transferred 0.5 mL aliquots of whole blood to 2 mL polypropylene containers; to obtain serum we centrifuged blood collected in red top tubes. CDC laboratories determined that specimen containers and syringes were lead free before the study. We stored anonymized frozen samples at −80°C except during transport in dry ice.

The CDC laboratories measured lead in whole blood using inductively coupled plasma mass spectrometry. This method has high accuracy and sensitivity with a limit of detection (LOD) of 0.25 μg/dL. Thirteen samples (4.6%) were below the LOD, which were replaced with 
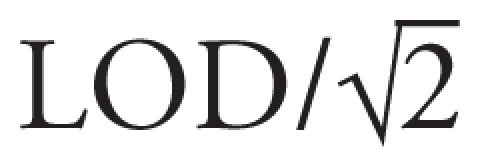
 for analyses. Because lead is readily transferred across the placental barrier, umbilical cord blood lead and maternal venous blood lead are highly correlated (Chuang et al. 2001), and cord blood lead levels can serve as a proxy measure for maternal blood lead levels in epidemiologic studies (Rabinowitz et al. 1987). CDC laboratories also measured cotinine in cord serum using liquid chromatography in conjunction with atmospheric pressure ionization mass spectrometry (LOD = 0.015 ng/mL) (Bernert et al. 1997). We classified a mother as a smoker if cord serum cotinine was ≥ 10 ng/mL (Pirkle et al. 1996) or if the medical record stated that she smoked at any time during pregnancy.

### Additional variables

We calculated prepregnancy body mass index (BMI) from prepregnancy weight and height recorded in the maternal medical record. For mothers with missing height (*n* = 6), prepregnancy weight (*n* = 3), or both (*n* = 1), we imputed values using regression models incorporating placental weight, weight gain during pregnancy, race, education, prepregnancy weight, and height. We also explored use of median imputation; results were similar to the multivariable regression (data not shown). Maternal medical records were also the source of information for maternal age, length of gestation, parity, anemia, and type of medical insurance.

For household income, we used U.S. Census 2000 median household income at the block group level. A block group was a subdivision of a census tract in which optimally 1,500 people reside but may range from 300 to 3,000. GeoLytics, Inc. (East Brunswick, NJ) matched 91% of maternal residences to block groups; we geocoded the remaining addresses using TerraServer USA (Microsoft Corporation, Redmond, WA) or ArcMap 9.0 with StreetMap 2000 (ESRI, Redlands, CA).

### Data analysis

We used Stata 9.3 (StataCorp LP, College Station, TX) for descriptive and multivariable regression analyses. A *p*-value < 0.05 was considered statistically significant. We used descriptive statistics to inform our unadjusted and adjusted multivariable regression analyses. Geometric means and 95% confidence intervals (CIs) are presented for lead because it has a log-normal distribution.

We evaluated several dose–response functions to describe the relationship of cord blood lead levels and blood pressure measures. We modeled lead as categorical, linear, logarithmic (natural), quadratic, and as a cubic spline. We compared these by examining lowess (nonparametric) smoothing curves, Akaike’s information criterion (AIC), Bayesian information criterion, adjusted *R*^2^ (or pseudo-*R*^2^) values, and root mean squared error. We selected lead quartiles as the model of choice. This option had a similar goodness of fit and has the added benefit of not limiting the exposure–outcome relationship to a specific form. Tests for trend are based on the *p*-value for the lead variable categorized into quartiles.

We evaluated additional variables for inclusion based on previous knowledge (Nawrot et al. 2002; Rothenberg et al. 2002) or because of associations with blood pressure measures and/or lead levels within this particular data set. These included maternal age, education, race, prepregnancy BMI, reported alcohol use during pregnancy, smoking status, parity, marital status, medical insurance type, neighborhood median household income, neighborhood per capita income, anemia, and gestational age. No relationship of umbilical cord blood lead with gestational age was evident in this data set (Spearman’s ρ = 0.04, *p* = 0.49).

We tested model assumptions and fit using quartile–quartile plots and residual versus fitted value plots. We performed sensitivity analyses to evaluate the effect of excluding the influential lead value (cord blood lead > 15 μg/dL) and the inclusion of imputed BMI values (data not shown). We assessed the possibility of effect modification with lead and BMI, maternal age, maternal race, or per capita income levels and saw no evidence of such effects.

To interpret results in a risk assessment context, we estimated benchmark dose (BMD) and the BMD lower confidence limit (BMDL) for the effect of lead exposure on maternal blood pressure using Benchmark Dose Software [version 2.1; developed by Lockheed Martin Corp. for the U.S. Environmental Protection Agency (U.S. EPA 2011)]. The BMD is an exposure level at which one observes a prespecified amount of response [e.g., the benchmark response (BMR)] in a population. The BMR (a health outcome) is typically a 5% or a 10% change in incidence (for dichotomous responses) or a 1-SD change in response (for continuous responses) compared with control individuals (U.S. EPA 2000). In this study, “control” individuals were defined as those with lead measurements in the lowest exposure quartile. The BMDL is the one-sided lower 95% confidence limit, equivalent to a 90% two-sided confidence limit. Currently, BMDLs are used by the U.S. EPA in some contexts as a point of departure for a risk assessment, in which uncertainty factors are added to calculate a regulatory or guidance level. This approach, and how it is an improvement over prior techniques, has been well described (Sand et al. 2008). One advantage of using such an approach for a substance such as lead is that, unlike a no observable adverse effect level (NOAEL), no assumption of a threshold for effects is required. However, this technique also has some drawbacks, including that for some outcomes it may be difficult to identify an appropriate critical effect size (e.g., BMR) (Dekkers et al. 2001; Travis et al. 2005) and for some data sets it is not possible to fit a BMD model (Travis et al. 2005).

Although originally designed to be used with toxicologic data, BMD methods have also been used with epidemiologic data (Budtz-Jorgensen et al. 2001). The most current version of the Benchmark Dose Software, however, does not yet allow for multivariable model fitting. Because several important risk factors other than lead exposure influence blood pressure levels, our BMD models incorporated these potential confounders by using adjusted blood pressure measurements obtained from our multivariate models (described above). We derived these by using linear combinations of model covariates (Stata’s lincom command), where we set each covariate to the study population mean. Our BMD models then consisted of lead quartiles as the exposure and adjusted maternal blood pressure as the response.

We used standard methods for determining a BMD (U.S. EPA 2000). The BMR was an increase of 1 SD of blood pressure from the blood pressure level among “controls” (those in the lowest quartile of umbilical cord lead concentrations). The 1-SD change generally approximates a 10% change (Crump 2002). We used the program’s default values for all other model parameters. We ran several different types of models (Hill, linear, polynomial, and power) and evaluated model fit using AIC and visual inspection.

## Results

Age at delivery ranged from 14 to 43 (mean = 26) years. There were 70.9% African Americans, 20.7% Caucasians, and 8.4% Asians. Fifty-four women (19.0%) were active smokers during pregnancy. Almost half (49.8%) were overweight or obese before pregnancy. In bivariate comparisons, lead levels were higher among Asians and African Americans compared with Caucasians, those living in neighborhood with a median income of < US$25,000 per year versus not, multiparous women, and those who smoked during pregnancy (Table 1).

At admission, mean SBP was 121.8 mmHg (95% CI, 119.9–123.7), mean DBP was 71.8 mmHg (70.4–73.1), maximum SBP was 144.3 mmHg (142.2–146.3), and maximum DBP was 77.4 mmHg (75.1–79.7). Sixteen women (5.6%) had GH or preeclampsia, and 27 women (9.5%) had any hypertensive condition. The geometric mean cord blood lead was 0.66 μg/dL (0.61–0.71 μg/dL), and the highest quartile for lead was 0.96–6.47 μg/dL.

We observed no increase in the odds of GH or preeclampsia or any hypertension related to increasing lead exposure levels (data not shown). However, mothers in the highest lead quartile had a statistically significant increase in admission and maximum SBP and DBP during labor and delivery compared with those in the lowest quartile, after adjusting for maternal age, maternal race, neighborhood median household income, primaparity, smoking during pregnancy, prepregnancy BMI, and anemia (Table 2). Specifically, we observed a 6.87-mmHg (95% CI, 1.51–12.21) increase in SBP and a 4.40-mmHg (0.21–8.59) increase in DBP admission blood pressure comparing the highest and lowest lead quartiles. Corresponding increases in maximum blood pressure were 7.72 mmHg (1.83–13.60) for SBP and 8.33 mmHg (1.14–15.53) for DBP.

We defined our BMR as a 1-SD increase in blood pressure; this was from 11.9 to 20.4 mmHg for all blood pressure outcomes. In comparing different types of BMD models, the power BMD models provided the best fit for the blood pressure measures overall (Table 3). Using this model, BMDs (BMDLs) were 1.46 μg/dL (1.42) for admission SBP, 1.91 μg/dL (1.43) for admission DBP, 1.45 μg/dL (1.41) for maximum SBP, and 1.47 μg/dL (1.43) for maximum DBP.

## Discussion

In this study we found a modest but significant association between very small increases in umbilical cord blood lead and elevations in SBP and DBP during labor and delivery. This association was observed at blood lead concentrations well below the current CDC recommended action level of 10 μg/dL for children and below the recommended level of 5 μg/dL for pregnant women (CDC 2007). Average blood lead concentrations in this study are lower than in prior studies of lead and blood pressure in pregnant women with the exception of the EDEN cohort study (Yazbeck et al. 2009).

Our findings are consistent with previous studies on blood lead levels and blood pressure during pregnancy (Magri et al. 2003; Rabinowitz et al. 1987; Rothenberg et al. 1999, 2002). Unlike other studies (Chen et al. 2006; Dawson et al. 2000; Magri et al. 2003; Sowers et al. 2002), we did not observe an association between lead and GH, preeclampsia, or clinically diagnosed hypertension as noted in the medical record. Except for that of Rabinowitz et al. (1987), these studies differed from ours in that they used maternal venous blood lead measurements and blood pressure taken before labor and delivery. Our measurements, blood pressure during labor and delivery as well as umbilical cord blood lead concentrations, have some limitations (as discussed below) but may still represent the relative lead exposure and blood pressure status of these women.

Studies of blood pressure customarily use the average of three measurements on a seated, resting subject. We did not collect, and therefore could not average, multiple blood pressure measurements for each individual; this may have introduced some nondifferential misclassification, which would be expected to attenuate results. Additionally, in our study, like Rabinowitz et al. (1987), we used blood pressure measurements taken during labor and delivery, a time of additional stress. Our blood pressure measures are therefore not equivalent to the standardized measures that one would obtain in a more controlled setting. However, because all of our blood pressure measures were taken during similar conditions for all women, they are likely to be similarly skewed. Supporting the validity of our blood pressure measures, women with previous hypertension diagnosis during or before pregnancy had higher admission and maximum blood pressure levels, and neither admission nor maximum pressures were correlated with the length of the hospital stay.

An important strength of this study was the quality of cord blood exposure measurements. Through obtaining clean samples and our use of high-quality analytic methods, we were able to obtain quantitative lead measurements for most of this population, despite the fact that their lead exposures were very low. Because of constraints of the study design, we were unable to measure maternal blood lead levels. Umbilical cord metal measurements, however, may serve as a proxy for maternal exposure. Indeed, umbilical cord blood lead is highly correlated with maternal venous blood lead [reported at *r*^2^ = 0.82 (Chuang et al. 2001)]; thus, use of cord blood lead will reasonably differentiate among different blood lead concentrations among mothers. Recognizing this, previous studies have also used cord blood lead to evaluate associations with maternal hypertension or preeclampsia (Rabinowitz et al. 1987; Vigeh et al. 2006). Several investigators report that umbilical cord blood lead concentrations are generally lower than maternal blood lead concentrations (Chuang et al. 2001; Harville et al. 2005; Rothenberg et al. 1996). In > 500 mother–infant pairs, the ratio of mean cord blood lead to mean maternal blood lead was 0.78 (Chuang et al. 2001). Assuming that this ratio holds in the present study, the geometric mean and 75th percentile of maternal blood lead would be approximately 0.86 and 1.24 μg/dL, respectively. These estimates for maternal venous blood in the present study are lower than concurrent estimates among adult women in the National Health and Nutrition Examination Survey, where geometric mean and 75th percentile were 1.22 and 1.80 μg/dL in 2003–2004 and 1.11 and 1.73 μg/dL in 2005–2006 (CDC 2010).

Bone lead is considered to be a better marker of long-term lead exposure, and blood lead a more precise estimate of short-term lead exposure (Hu et al. 2007). However, during pregnancy there is potentially a greater release of lead from bone into blood (Gulson et al. 1997), and maternal blood may be derived from mobilization of lead in bone. Nevertheless, if the mechanism(s) by which lead affects maternal blood pressure levels is heavily influenced by long-term lead exposure, bone lead could be a better biomarker for describing a relationship of lead exposure with blood pressure. Rothenberg et al. (2002) found associations of maternal hypertension and elevations in blood pressure levels with maternal bone (calcaneous) lead but not with maternal venous blood lead. On the other hand, short-term or transient lead effects on blood pressure might be better assessed with biomarkers of recent lead dose such as blood lead (Navas-Acien et al. 2008). Knowledge of the mechanism by which lead exerts effects on blood pressure would be helpful in determining whether blood or bone lead is a better biomarker for studying this relationship; although mechanisms have been suggested (Vaziri 2008), more work is ongoing.

Although maternal venous and umbilical cord blood lead are highly correlated, this correlation is not equivalent. If factors influencing the ratio of maternal blood lead to cord blood were not related to blood pressure, this would result in random misclassification of exposure, and as a result, our findings would be biased toward the null. In contrast, Harville et al. (2005) reported that higher maternal blood pressure is related to a lower ratio of maternal blood lead to cord blood lead; in that situation, our results could have been biased away from the null. However, evidence supporting this conclusion is somewhat limited because Harville et al. (2005) observed this relationship only in the subset of women > 30 years of age or with > 40 pounds of weight gain. Meanwhile, our findings are consistent with prospective evidence among nonpregnant adults, where placental transfer of lead would not influence results (Glenn et al. 2003), providing additional support for an association between lead exposure and subsequent elevations in blood pressure.

Prior work has evaluated the influence of measurements of hematocrit or hemoglobin, stress, calcium, or zinc on the relationship between lead exposure and blood pressure levels (Hense et al. 1993; Nawrot et al. 2002; Peters et al. 2007; Rabinowitz et al. 1987; Rothenberg et al. 1999). These data were not available for this study. Although hematocrit and hemoglobin have been associated with low lead concentrations (Harville et al. 2005), their role as a confounders has been proposed to be more important among older women (> 50 years) (Hense et al. 1993). Moreover, the association between blood lead levels and GH was only slightly decreased and remained statistically significant after further adjustment for hematocrit in a study during the third trimester of pregnancy (Magri et al. 2003). Stress has been identified as an effect modifier of the lead–blood pressure relationship; those with higher stress had a higher risk of developing hypertension after lead exposure (Peters et al. 2007). The observation that women in our population with higher cord blood leads had higher maximum blood pressure levels during labor and delivery is consistent with that work.

Our BMD analysis of these data provides a different perspective of the relationship we observed between lead exposure and blood pressure increase. In terms of estimated maternal blood lead levels from cord blood levels (calculated as described above), we found a BMDL of roughly 1.4 μg/dL venous blood lead for all blood pressure outcomes. The BMDL, again, is an exposure level that, with 95% confidence, corresponds to the prespecified response (1 SD of blood pressure) (Sand et al. 2008). Although BMD analysis is frequently used in the process of creating regulatory exposure standards, our use of it here should not be interpreted in a regulatory context, but rather as a description of an exposure level in this study where we observed measurable effects.

No threshold effect of lead has been identified. Action levels for lead are based in part on our ability to create and invest in effective interventions for lead exposure, while recognizing that the goal is to reduce exposures to the greatest extent possible (Bernard 2003). There is initial evidence that calcium supplementation may be related to lower blood lead levels in pregnancy by reducing the release of lead from bone lead stores (Ettinger et al. 2009), although this needs replication. Better still would be primary prevention, or preventing lead exposure in the first place; this could occur, for example, by limiting the amount of lead released to the environment by implementing safer work practices (U.S. EPA 2010).

In this work, we show that the previously established association of blood lead levels with elevations in blood pressure can be replicated among pregnant women at the time of labor and delivery, extending previous findings to even lower lead exposure levels. Because even small reductions in population blood pressure could result in substantial public health benefits (Whelton et al. 2002), this work suggests that continued reductions in lead exposure remain an important public health goal.

## Figures and Tables

**Table 1:** Geometric mean and 95% CIs for umbilical cord lead concentrations (μg/dL) by population characteristics: Baltimore THREE Study, 2004–2005 (*n* = 285).

Characteristic	*n*	Geometric mean	95% CI
All mothers	285	0.66	0.61–0.70

Age, years			
< 20	58	0.72	0.64–0.81
20–30	134	0.64	0.57–0.71
> 30	93	0.64	0.56–0.73

Race			
Caucasian	59	0.44*	0.38–0.52
Asian	24	0.85*	0.67–1.09
African American	202	0.72*	0.66–0.77

Median household income (US$)			
< 25,000	89	0.81*	0.71–0.92
25,000–50,000	151	0.64*	0.59–0.70
> 50,000	45	0.55*	0.50–0.61

Parity			
Primiparous	118	0.61*	0.55–0.67
Multiparous	167	0.69*	0.63–0.76

Smoking			
Nonsmoker	231	0.61*	0.57–0.67
Active smoker	54	0.83*	0.71–0.97

Prepregnancy BMI (kg/m^2^)			
< 18.5	14	0.58	0.42–0.80
18.5–24.9	129	0.63	0.57–0.70
25.0–29.9	71	0.66	0.57–0.76
≥ 30	71	0.72	0.62–0.82

Anemia			
Not anemic	249	0.65	0.60–0.70
Anemic	36	0.69	0.58–0.80

Admission SBP (mmHg)			
< 140	252	0.65	0.60–0.70
≥ 140	33	0.73	0.60–0.89

Admission DBP (mmHg)			
< 90	265	0.65	0.60–0.70
≥ 90	20	0.74	0.60–0.91

Maximum SBP (mmHg)			
< 140	123	0.60*	0.54–0.67
≥ 140	162	0.70*	0.63–0.77

Maximum DBP (mmHg)			
< 90	229	0.63*	0.58–0.68
≥ 90	56	0.78*	0.67–0.92

* Statistically significant difference in geometric mean lead levels across categories, *p* < 0.05, one-way ANOVA or Student’s *t*-test.

**Table 2:** Change in blood pressure (mmHg) by quartiles of cord blood lead exposure based on multivariable regression models: Baltimore THREE Study, 2004–2005 (*n* = 285).

Parameter	First quartile	Second quartile	Third quartile	Fourth quartile	*p*-Trend
*n*	72	72	71	70	
Lead (μg/dL)	≤ 0.46	0.47–0.65	0.66–0.95	≥ 0.96	
Admission					
SBP	Referent	2.89 (−2.16 to 7.94)	1.05 (−4.04 to 6.14)	6.87 (1.51 to 12.21)*	0.033*
DBP	Referent	0.00 (−3.95 to 3.96)	0.81 (−3.17 to 4.80)	4.40 (0.21 to 8.59)*	0.036*
Maximum					
SBP	Referent	2.47 (−3.08 to 8.02)	−1.76 (−7.36 to 3.85)	7.72 (1.83 to 13.60)*	0.055
DBP	Referent	3.93 (−2.86 to 10.72)	−0.42 (−7.27 to 6.43)	8.33 (1.14 to 15.53)*	0.086

Multivariable linear regression models were controlled for maternal age, maternal race, neighborhood median household income, primaparity, smoking during pregnancy, prepregnancy BMI, and anemia. *p*-Trend is based on a quartile model.

* *p* < 0.05.

**Table 3:** BMD values for umbilical cord blood lead (μg/dL) and maternal blood pressure, comparing Hill, linear, polynomial, and power models.

Measurement	Hill	Linear	Polynomial	Power
Admission SBP				
BMD	1.50	2.57	0.22	1.46
BMDL	1.42	1.57	0.07	1.42
AIC	1,840	1,838	1,840	1,838

Admission DBP				
BMD	NC	2.71	NC	1.91
BMDL	NC	1.62	NC	1.43
AIC	NC	1,698	NC	1,699

Maximum SBP				
BMD	1.46	2.50	0.15	1.45
BMDL	1.41	1.55	0.06	1.41
AIC	1,895	1,897	1,895	1,893

Maximum DBP				
BMD	1.50	2.95	0.15	1.47
BMDL	1.43	1.71	0.06	1.43
AIC	2,009	2,009	2,009	2,007

NC, no calculation. BMD values were generated with Benchmark Dose Software using the geometric mean for umbilical cord blood and adjusted maternal blood pressure (derived from models presented in Table 2) for each lead quartile. Average SDs for blood pressure were 15.1 mmHg (SBP admission), 11.9 mmHg (DBP admission), 16.7 mmHg (SBP maximum), and 20.4 mmHg (DBP maximum). The AIC is a unitless measure of model fit.
